# Isolation of Porcine Umbilical Cord Cells by Mechanical Tissue Dissociation Using a Tissue Grinder

**DOI:** 10.3390/cells14181425

**Published:** 2025-09-11

**Authors:** Katja Stange, Tessa Wolter, Zhenpei Fu, Gracie Burdeos, Yonatan Mideksa, Andreas Friese, Monika Röntgen

**Affiliations:** 1 Research Institute for Farm Animal Biology (FBN), Wilhelm-Stahl-Allee 2, 18196 Dummerstorf, Germany; stange.katja@fbn-dummerstorf.de (K.S.); tessa.wolter@med.uni-rostock.de (T.W.); zf2024.work@gmail.com (Z.F.); burdeos@fbn-dummerstorf.de (G.B.); 2 OMNI Life Science GmbH & Co. KG, Karl-Ferdinand-Braun-Strasse 2, 28359 Bremen, Germany; ymideksa@ols-bio.de (Y.M.); andreas.friese@ols-bio.de (A.F.)

**Keywords:** tissue grinding, umbilical cord, MSC, multipotency

## Abstract

Primary cells better reflect the physiological situation, and mesenchymal stromal cells (MSCs), especially, are promising candidates for biomedical applications. MSCs from the umbilical cord (UC) can be collected easily, non-invasively, and painlessly and do not involve ethical problems. The derived cell products harbor great potential in stem cell technology and agricultural applications. A tissue grinder (TIGR) was used to homogenize porcine UC tissue and to dissociate the UC cells, thereby testing different tissue-to-medium ratios. Cells were cultivated until passage 3, and the proliferation rate, metabolic activity, colony forming ability, surface marker expression, and multi-lineage differentiation potential were assessed. Tissue grinding could be successfully used to isolate UC-derived porcine cells with a high yield and viability, as well as an increasing proliferation rate during cultivation. Isolated cells showed MSC-like features: the expression of CD73, CD90, and CD105, ability to form colonies, and adipogenic, chondrogenic, and osteogenic differentiation. Tissue grinding is highly suitable for isolating high-quality cells from whole UC tissue of pigs in a fast and reproducible way. Cells might be used in a wide range of therapeutical and biotechnological applications, such as understanding and treating severe disorders, drug screening, or tissue engineering. Cells from supposedly waste tissues like UC will be especially useful in transplantation medicine.

## 1. Introduction

Cell cultures are important tools for investigating cell and tissue behavior and function in vitro and are also utilized for the production of biologicals such as antibodies or vaccines. Although permanent, indefinitely proliferating cell lines like Chinese hamster ovary (CHO), human embryonic kidney (HEK), Jurkat, or C2C12 have been established for those purposes, working with primary cells is inevitable and very common for other applications [1]. Primary cells are obtained directly from the tissues or organs of an organism. Due to their limited life span, they better reflect the physiological situation and show a superior maintenance of functionality and other characteristics, e.g., cell-type-specific marker expression, compared to permanent cell lines [2,3]. Thus, for tissue replacement and gene therapy, primary stem cells, particularly embryonic stem cells and induced pluripotent stem cells, are used. However, in cell therapy, both cell types have become problematic because of ethical concerns and reported safety problems like teratoma formation [4,5,6,7].

Because of these limitations, mesenchymal stromal cells (MSCs) from several adult and perinatal tissues have aroused greater interest. MSCs are multipotent, show a spindle-shaped morphology, and have the ability to self-renew [6]. For non-human species, no specific definition for stem cell populations or MSCs exists. Thus, as an orientation, we used the minimal criteria of the International Society for Cell and Gene Therapy (ISCT) which describe the characteristics of in vitro-expanded MSCs of human origin. They include plastic adherence under standard culture conditions, the capacity for osteogenic, adipogenic, and chondrogenic differentiation, the positive expression of CD73, CD90, and CD105 surface antigens (≥95% of the cell population), but the absence (≤2% of the cell population) of the hematopoietic and endothelial lineage markers CD11b, CD14, CD19, CD34, CD45, CD79a, and HLA-DR [8]. The considerable differentiation and regeneration potential of MSCs in conjunction with positive paracrine effects on proliferation and angiogenesis as well as anti-inflammatory, antiapoptotic, and immunomodulatory properties made them promising candidates for biomedical applications [9,10]. Indeed, MSCs or their secretions have been tested as a treatment for various human and animal clinical conditions [4,5,6,7,11,12,13,14,15]. In addition, MSCs are promising candidates for use in cultured meat production [16].

Although MSCs reside in and can be isolated from various tissues, the most commonly used sources are bone marrow, followed by adipose tissue and umbilical cord (UC) [17,18]. However, Wang et al. [19], by using single-cell RNA sequencing, showed that MSCs from different tissues (bone marrow, adipose, dermis, and UC) have striking transcriptomic and, thus, functional heterogeneity. In UC-MSC-specific subpopulations, genes related to anti-inflammation and anti-aging were highly upregulated. In accordance to this, UC-derived MSCs have active telomerase and express pluripotency genes such as *SOX 2* and *OCT 4,* resulting in enhanced proliferation activity and self-renewal potential compared with adult MSCs [20,21,22,23,24]. Studies have also shown that UC-MSC are superior to other MSCs in differentiating into tendon (in vitro and in vivo) [25]. In addition, umbilical cords can be collected easily, non-invasively, and painlessly and do not involve ethical problems [6,7,21].

The MSC containing connective tissue of the UC develops by the fusion of the body stalk and the amnion mesoderm. This specialized, mucous connective tissue, the so-called Wharton’s Jelly (WJ), is mainly composed of proteoglycans, hyaluronic acid, collagen, and elastic and reticular fibers, and covered by a monolayer epithelium of ectodermal origin [7,26]. The significant intra-tissue heterogeneity of UC-MSC subpopulations is mainly related to genes (*DPT*, *Col15A1*, *CLDN11*, and *TIMP3*) of this extracellular matrix (ECM) [19]. According to the cytoskeletal complexity, the most immature cells are located in subamniotic and intervascular regions, whereas cells of perivascular regions may represent a more differentiated state [27,28]. Depending on the species, the UC comprises three (human and pig: two arteries and one vein) to four blood vessels (cattle, sheep, goat, alpaca, buffalo, canine, and feline: two arteries and two veins) [26,29,30,31]. Cells from whole UC or specific UC compartments (as WJ, blood vessels, and UC membrane) were successfully isolated from different species, e.g., human [7,21,22,32], equine [33], bovine [34], buffalo [35], or pig [36,37]. However, species-dependent differences in the anatomy of the UC, particularly the umbilical cord vessels, can influence the type and characteristics of cellular populations [28,29]. In addition, UC-MSC from animals are not characterized to the extent of human UC-MSC. Thus, the human criteria for identifying and characterizing those cells in animals need to be evaluated closely.

Here, we use porcine material, which offers many benefits regarding tissue sampling and applications. Compared to other animals such as mouse or rat, pig organ physiology shows more similarity to that of humans, making it a better experimental model for testing biomedical applications and evaluating the choice of cells for their development [38]. Being a polytocous species, more grafts can be obtained from one sow at birth in a batch manner [37]. The derived cell products harbor great potential in human stem cell technology and, specifically, in cellular agricultural applications [22,39]. Using xenogeneic products comes with some concerns and is still in its infancy. But, in organ transplantation, it has already achieved amazing milestones [40,41] and might be inevitable in order to overcome the organ transplant shortage [37].

The isolation method has a high impact on the cell yield and quality (e.g., viability, and ability to proliferate and differentiate) of primary cells like UC-MSC and, thus, determines the reliability of the results [26]. In general, either explant cultures or dissociation methods (or a combination) are used, both having advantages and drawbacks [1], and both procedures can be performed with or without discarding the cord vessels. In the enzymatic method, chopped pieces of UC tissue are digested using collagenase, hyaluronidase, trypsin, or other enzymes [42,43]. A cell suspension can be obtained quicker than via explant cultures, but the over-digestion of tissue can result in a lower cell viability, degradation of cellular surface proteins, and negative effects on the ability to proliferate and differentiate [44,45,46]. Besides ethical and animal welfare concerns, animal-derived enzymes like Trypsin can lead to the contamination of cell cultures, e.g., due to undetected viruses [47,48]. In explant cultures, small tissue pieces, which are mostly cut manually, are placed on a culture dish to allow migration and the adherence of cells to the surface [7,35]. Besides its simplicity [27], the explant method provides a better yield and viability of MSCs, with a higher and more homogeneous number of MSC-like cells [45,46]. However, the autonomous migration of cells might favor cell types or subpopulations with a higher migration potential, while others are lost [44]. In addition, this procedure is more time-consuming because about two weeks are needed until P0 is finished [21,27].

In order to optimize, standardize, and shorten the cell-sourcing procedure, specifically if larger quantities need to be processed, (semi-)automated devices might be very useful [1]. To this end, tissue grinders can greatly help to homogenize the tissue and to dissociate cells. The sample is put between two hard surfaces, which slide against each other [49]. Here, we used the TIGR device (OMNI Life Science) with two counter-rotating rows of grinding teeth [50]. The procedure was described for cell isolation from the lung, liver, kidney, spleen, thymus, colon, intestine, pancreas, stomach, and lymph node [44,50]. The isolation of umbilical cord cells (UCCs) by using this tissue-grinding procedure has not been described before. Here, we tested the feasibility of this method using porcine UC tissue and performed a first characterization of the isolated porcine UCC (pUCC).

## 2. Materials and Methods

### 2.1. Pig Serum Production

Whole-blood samples from male and female slaughter pigs from German Landrace (body weight 25–81.5 kg, age 81–138 days) were received from the experimental abattoir of the Research Institute for Farm Animal Biology (FBN), approved by the E.U. and the German quality management system QS. Slaughtering was performed according to the guidelines of the Animal Care Committee of the State Mecklenburg-Western Pomerania, Germany, based on the German Law of Animal Protection. Serum was obtained by centrifugation of coagulated whole-blood samples and sterilized by sterile filtration through a 0.2 µm filter (Sarstedt, Nümbrecht, Germany) and subsequent UV treatment. Before use, the serum from 18 animals was pooled to minimize variability due to animal-specific differences.

### 2.2. Isolation and Cultivation of Umbilical Cord Cells (UCCs)

**Figure 1:**
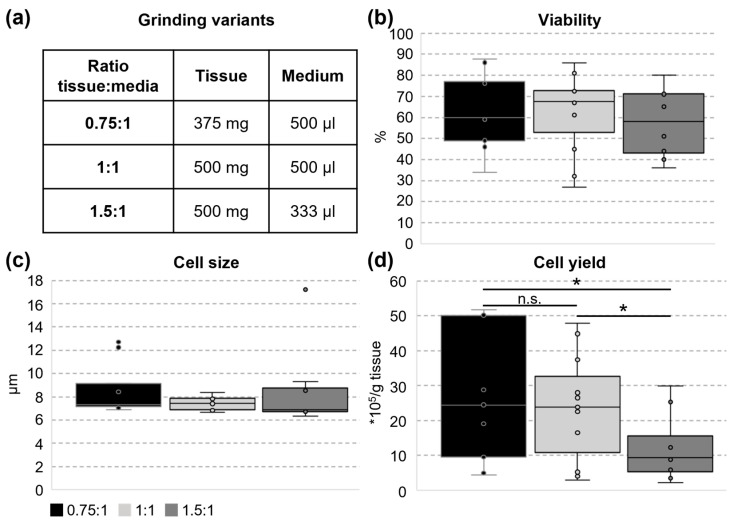
Cellular parameters directly after tissue grinding. Umbilical cord cells (UCCs) were isolated by tissue grinding, and cell viability, size, and yield were determined directly afterwards (0.75:1 n = 9, 1:1 n = 11, and 1.5:1 n = 8). (**a**) Three experimental conditions were compared, differing in the amounts of tissue and medium added to the grinding unit. (**b**) Cell viability showed no difference between the tested variants. One-Way Analysis of Variance was used for statistical analysis. (**c**) For cell size, no difference between tested variants was detected. Kruskal–Wallis One-Way Analysis of Variance on Ranks was used for statistical analysis. (**d**) The cell yield of the variants 0.75:1 and 1:1 did not differ from each other, whereas the number of cells isolated per gram tissue was significantly lower for condition 1.5:1. Kruskal–Wallis One-Way Analysis of Variance on Ranks was used for statistical analysis (* *p* ≤ 0.05, n.s. not significant).

Umbilical cords (UCs) from newborn German Landrace piglets were collected when delivered from the experimental pig unit of the Research Institute for Farm Animal Biology (FBN). No animal experiment was conducted, since there was no intervention in the process of birth itself (no ethical approval needed). In total, 24 umbilical cords from female and male piglets were used within approximately 1–3 h after sampling for the shown experiments. UC were washed in 0.9% NaCl solution containing 100 U/mL Penicillin, 100 µg/mL Streptomycin, and 2.5 µg/mL Amphotericin B (all from PAN Biotech, Aidenbach, Germany). In a sterile petri dish, 2 cm long pieces of UC were cut longitudinally to remove blood vessels as described in [22]. The remaining tissue was cut into small pieces (maximum area 0.25 cm^2^), weighed, and incubated in inoculation medium (alphaMEM containing 20% fetal bovine serum (FBS, PAN Biotech) and 100 µg/mL Primocin (Invivogen, Toulouse, France) for 20 min. For single cell isolation, the TIGR Tissue Grinder & Dissociator (Omni Life Science GmbH, Bremen, Germany) with a grinder unit containing a 100 µm cell strainer and the pre-installed program “medium” was used (step 1: cutting 30 s at 70 rpm, step 2: grinding 30 s at 70 rpm, step 3: cutting 25 s at 50 rpm, and step 4: cutting 25 s at 50 rpm; [44]). Different ratios of tissue (375 or 500 mg) and medium (333 or 500 µL) were tested, as shown in Figure 1a. After centrifugation (500 g, 10 min, and 10 °C), cell viability, size, and number were determined using Countess automated cell counter (Thermo Fisher, Darmstadt, Germany). The cells obtained from each of the four grinder units were seeded in a 6 cm cell culture dish (Sarstedt, Nümbrecht, Germany, mean seeding density: 53,772 cells/cm^2^) in inoculation medium as passage 0 and incubated at 37 °C with 5% CO_2_ in a humidified atmosphere. After 24 h, medium was changed to initial growth medium (alphaMEM containing 10% FBS and 100 µg/mL Primocin). Subsequently, medium was changed twice a week during further cultivation using growth medium (alphaMEM containing 10% FBS, 100 U/mL Penicillin, 100 µg/mL Streptomycin, and 2.5 µg/mL Amphotericin B). After approximately 1 week of cultivation, 400 µL of aspirated medium were plated on 4% CASO Agar plates (Carl Roth, Karlsruhe, Germany) to evaluate sterility of cell cultures. No bacterial colonies were seen after 3 days of incubation at 37 °C for all used UC cell cultures. After 19 days, UC cells were passaged for the first time using 0.05% trypsin/0.02% EDTA (PAN Biotech). For further passages, cells were seeded at a desired density of approximately 5000 cells/cm^2^ (in mean 4825.5 ± 428.6 cells/cm^2^) and passaged weekly. Brightfield images of cultivated cells were acquired after 15 days of cultivation (passage 0) or 1 day before passaging (passage 1, 2, and 3) with the microscope Primovert KMAT (Carl Zeiss, Oberlochen, Germany) and the corresponding camera Axiocam 208 color. Representative images are shown.

### 2.3. Surface Marker Analysis

Directly after the first harvest (day 19), cells were resuspended in PBS containing 0.5% bovine serum albumin (Sigma-Aldrich, Taufkirchen, Germany) and 2 mM EDTA. Subsequently, antibodies against CD105 (PE-conjugated, clone MEM-229, Sigma-Aldrich, Taufkirchen, Germany), CD90 (FITC-conjugated, clone 5E10, Thermo Fisher Scientific, Schwerte, Germany), and CD73 (unconjugated, clone 1F22, Sigma-Aldrich, Taufkirchen, Germany) were added and incubated for 1 h. Isotype controls for CD105 (Merck, Darmstadt, Germany) and CD90 (Thermo Fisher Scientific, Schwerte, Germany) were incubated in a separate sample; secondary antibody only was used as a staining control for CD73. After washing with PBS, the secondary goat anti-rabbit antibody (Alexa Fluor 647, Thermo Fisher Scientific, Schwerte, Germany) was incubated with the cells for 1 h. After washing again with PBS, cells were separated via a 50 µm cell strainer (Sysmex, Norderstedt, Germany) and analyzed (10,000 events per sample) by using an argon-equipped Gallios Flow Cytometer (Beckman Coulter, Krefeld, Germany) and evaluated with Kaluza software version 2.1.2.20011 (Beckman Coulter, Krefeld, Germany).

### 2.4. CFU Assay

For colony-forming unit (CFU) assay, cells in passage 1 and 3 were seeded in growth medium at a density of 200 cells/well in a 6-well plate (Sarstedt, Nümbrecht, Germany). Medium was changed twice a week. After 14 days, cells were fixed with 4% paraformaldehyde and stained with 0.5% crystal violet (Carl Roth, Karlsruhe, Germany). Colonies containing more than 50 cells were counted manually.

### 2.5. Metabolic Activity (WST1)

To assess the metabolic activity of isolated cells, cells were seeded in a 96-well plate (1600 cells/well) in passage 1 and 3 using growth medium. After 3 and 7 days of cultivation, the supernatant was used to perform WST-1 assay according to the manufacturer’s instructions (Merck, Darmstadt, Germany). In addition, cells were stained with 0.5% crystal violet before bound dye was solubilized again with methanol to measure absorbance at 570 nm.

### 2.6. Multi-Lineage Differentiation Assay

For adipogenic differentiation, 0.1 × 10^5^ cells/well were seeded in a 24-well plate (Sarstedt) in passage 3 using growth medium. After 2 days, 70–80% confluency was reached and medium was changed to induction medium (DMEM 4.5 g/L glucose (PAN Biotech, Aidenbach, Germany) containing 5% pig serum, 60 µM Indomethacin (Sigma-Aldrich, Taufkirchen, Germany), 10 µg/mL rh Insulin (Biozym, Hessisch Oldendorf, Germany), 100 U/mL Penicillin, 100 µg/mL Streptomycin, and 2.5 µg/mL Amphotericin B). One week after induction, 1 µM Dexamethasone (Thermo Fisher Scientific, Schwerte, Germany) and 0.5 mM 3-isobutyl-1-methylxanthine (IBMX, Sigma-Aldrich, Taufkirchen, Germany) were added into the medium (differentiation medium). Media were changed twice a week. After 11 days of differentiation, lipid droplets were stained with Oil Red O. Therefore, cells were fixed with 4% paraformaldehyde, permeabilized with 60% isopropanol, and incubated with 0.2% Oil Red O (Merck, Darmstadt, Germany). Brightfield images of Oil Red O staining were acquired with the microscope Leica DM4000B (Leica, Wetzlar, Germany) and the corresponding software (cellSens 3.2, Olympus, Tokyo, Japan).

For chondrogenic differentiation, cells were used for micromass culture in passage 3. Therefore, 2.4 × 10^5^ cells were seeded in 12 µL chondrogenic medium (DMEM 1 g/L glucose (PAN Biotech, Aidenbach, Germany), 10% FBS, 100 U/mL Penicillin, 100 µg/mL Streptomycin, and 2.5 µg/mL Amphotericin B) as a small drop in the middle of the well of a 24-well plate (Sarstedt, Nümbrecht, Germany). The cultures were incubated for 1 h to allow cells to settle before 1 mL chondrogenic medium was added. Cells were cultivated for 14 days and medium was changed twice a week. Micromass cultures were fixed in Kahles fixative (28.9% ethanol, 0.37% formaldehyde, and 3.9% acetic acid in H_2_O) and subsequently stained with 0.05% Alcian Blue 8GX (Thermo Fisher Scientific, Schwerte, Germany) in 0.1M HCl. Images of Alcian-Blue-stained cultures were acquired with the camera Sony RX100 M3 (Sony, Tokyo, Japan).

For osteogenic differentiation, 0.5 × 10^5^ cells/well were seeded in a 24-well plate coated with Collagen I (Greiner bio-one, Frickenhausen, Germany) in passage 4 using osteogenic growth medium (DMEM 1 g/L glucose, 10% FBS, 100 U/mL Penicillin, 100 µg/mL Streptomycin, and 2.5 µg/mL Amphotericin B). After reaching confluency, medium was changed to osteogenic differentiation medium (DMEM 1 g/L glucose, 10% FBS, 100 nM Dexamethasone (Thermo Fisher Scientific, Schwerte, Germany), 10 mM β-glycerol phosphate (Carl Roth, Karlsruhe, Germany), 50 ng/mL vitamin C (Sigma-Aldrich, Taufkirchen, Germany), 100 nM vitamin D (Biomol, Hamburg, Germany), 100 U/mL Penicillin, 100 µg/mL Streptomycin, and 2.5 µg/mL Amphotericin B) as described in [37]. Cells were allowed to differentiate for 14 days with medium change twice a week, fixed with 4% paraformaldehyde, and stained with 0.5% Alizarin Red S (Santa Cruz Biotechnology, Heidelberg, Germany). Brightfield images of Alizarin Red staining were acquired with the microscope Leica DM4000B (Leica, Wetzlar, Germany) and the corresponding software (cellSens 3.2, Olympus). For all differentiation assays, representative images are shown.

### 2.7. Statistical Analyses

For statistical analysis, SigmaPlot 13.0 (Systat Software Inc., San Jose, CA, USA) was used. Data are shown as mean ± SD. If Normality Test (Shapiro–Wilk) and Equal Variance Test (Brown–Forsythe) were passed, One-Way Analysis of Variance was performed. Otherwise, Kruskal–Wallis One-Way Analysis of Variance on Ranks was performed. Box-and-whisker plots are presented with the maximum 1.5 of the interquartile range (Q1–Q3). Outliers were excluded from statistical analyses and statistical significances were assigned as follows: *** *p* ≤ 0.001, ** *p* ≤ 0.01, * *p* ≤ 0.05, trend (T) *p* ≤ 0.1, and n.s. not significant.

## 3. Results

### 3.1. Isolation of UC Cells Comparing Different Tissue-Grinding Methods

The TIGR system was used for tissue grinding in order to isolate MSC-like cells from porcine umbilical cords. The ratio of tissue to medium varied between variants (0.75:1, 1:1, and 1.5:1; Figure 1a) while the grinding profile was kept constant. The obtained cell suspension was analyzed immediately after isolation to assess the cell viability, size, and number (Figure 1b–d). The viability of cells was not influenced by the tissue-to-medium ratio and, in mean, 61.2% ± 17.9 of all cells were viable directly after grinding. The cell size did also not differ between the tested variants and showed a quite low variability. With 27.2 × 10^5^ ± 19.7 cells per g tissue, the best cell yield could be achieved by using a 0.75:1 tissue-to-medium ratio. Due to the biological variance between different animals, the cell yield of variant 1:1 was not significantly lower, but yielded 13% fewer cells (23.6 × 10^5^ ± 15.7 cells/g tissue). When using a 1.5:1 tissue-to-medium ratio, only 45% of the cell yield of variant 0.75:1 could be reached (12.2 × 10^5^ ± 10.1 cells/ g tissue), which represents a statistically significant loss of cells.

### 3.2. Cultivation and Proliferation of UC Cells

**Figure 2:**
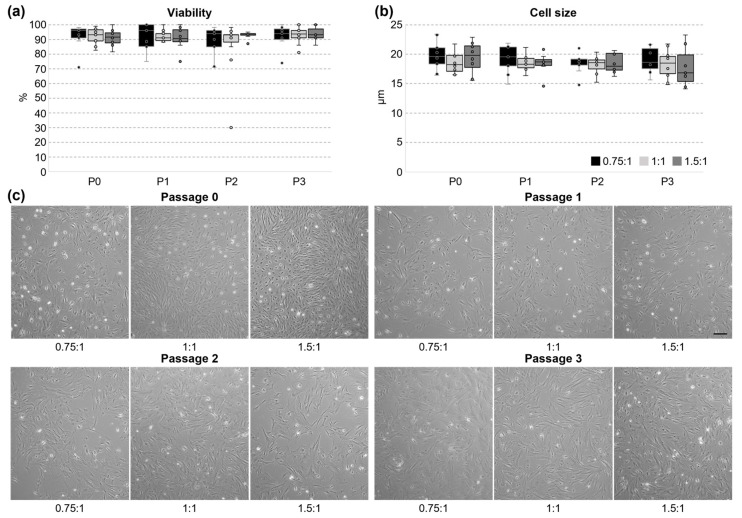
Cultivation of UC cells. UC cells were cultivated until passage 3 (0.75:1 n = 9, 1:1 n = 11, and 1.5:1 n = 8). (**a**) The cell viability showed no significant differences between passages or tested variants. Kruskal–Wallis One-Way Analysis of Variance on Ranks was used for statistical analysis (all not significant). (**b**) The size of cultivated cells remained stable over four passages and showed no significant difference between tested variants. One-Way Analysis of Variance was used for statistical analysis (all not significant). (**c**) Cellular morphology of isolated cells was monitored from passage 0 until the end of passage 3 (scale bar 200 µm).

Isolated UC cells were seeded on cell culture dishes and cultivated until passage 3 to further observe the cellular properties. The viability of the cells was very good throughout all the tested passages (overall mean 91.1% ± 8.9) and no statistical differences were observed (Figure 2a). The cell size remained constant between passages and tested variants, as well (Figure 2b). The mean cell size during cultivation was 18.6 ± 2.1 µm.

Before the first dissociation, cells were small and the morphology changed from round/spherical, to polygonal and spindle-shaped (Figure 2c). Some cells started to spread out already. Only a small amount of red blood cells was seen and could be easily removed by washing during the first days in the culture. From passage 1 on, cells became more homogenous and larger, and there were more cells spread out on the surface. In passage 3, some cells were slightly elongated, but showed no branching. Overall, only an insignificant number of floating cells was seen, reflecting the high viability. The cell morphology did not depend on the chosen grinding variant during isolation.

**Figure 3:**
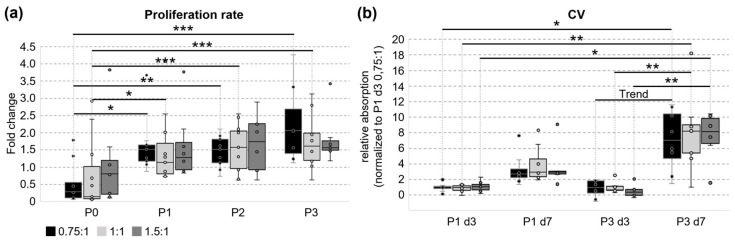
Proliferation of UC cells comparing different grinding variants. (**a**) The proliferation rate was calculated as the ratio of the cell number at the end and at the beginning of each passage, respectively (0.75:1 n = 9, 1:1 n = 11, and 1.5:1 n = 8). The proliferation significantly accelerates for variant 0.75:1 and 1:1 during cultivation. One-Way Analysis of Variance was used for statistical analysis (*** *p* ≤ 0.001, ** *p* ≤ 0.01, and * *p* ≤ 0.05). Only significant differences within the same condition (between different time points) or within the same time point (between different conditions) are shown. (**b**) Cells grown in a 96-well plate were stained with crystal violet (CV) to monitor cell growth in passage 1 and 3 (0.75:1 n = 8, 1:1 n = 9, and 1.5:1 n = 7). No significant differences were seen between tested variants. The values for each tested biological replicate were normalized to the mean for all samples from variant 0.75:1 in P1 at day 3. Kruskal–Wallis One-Way Analysis of Variance on Ranks was used for statistical analysis (** *p* ≤ 0.01, * *p* ≤ 0.05, and Trend *p* ≤ 0.1). Only significant differences within the same condition (between different time points) or within the same time point (between different conditions) are shown.

Cellular growth was monitored during cultivation. Firstly, the proliferation rate was calculated from the number of cells which were seeded with equal cell density and harvested during passaging (Figure 3a). Using the grinding variant 0.75:1 and 1:1, the proliferation was significantly enhanced in all later passages compared to P0. Between passage 1, 2, and 3, no difference was seen anymore within the tested variants. In contrast, when using relatively more tissue than medium during grinding (1.5:1), later passages did not proliferate faster than the initial cell culture since no significant improvement of proliferation was seen during cultivation. Nevertheless, the proliferation rate revealed no significant differences between the tested variants.

Secondly, cells were seeded with similar densities in passage 1 and 3, and cellular components were stained with crystal violet after 3 or 7 days of incubation (Figure 3b). The amount of incorporated dye was quantified afterwards to complement the analysis of the proliferation rate. In contrast to passage 1, a significant increase in cellular growth was detected between day 3 and day 7 in passage 3 for all grinding variants. Similar to the proliferation rate, no significant differences between variants were detected.

### 3.3. Metabolic Activity and Ability to Form Colonies

**Figure 4:**
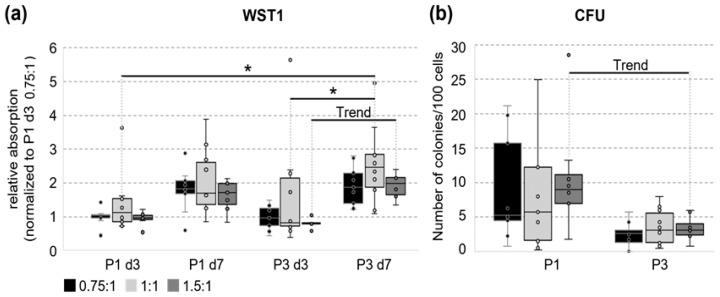
Metabolic activity and colony-forming ability of UC cells. (**a**) The metabolic activity of isolated cells was tested using WST-1 assay in passage 1 and 3 (0.75:1 n = 9, 1:1 n = 10, and 1.5:1 n = 7). No significant differences between tested variants were detected. The values for each tested biological replicate were normalized to the mean for all samples from variant 0.75:1 in P1 at day 3. Kruskal–Wallis One-Way Analysis of Variance on Ranks was used for statistical analysis * *p* ≤ 0.05, and Trend *p* ≤ 0.1). Only significant differences within the same condition (between different time points) or within the same time point (between different conditions) are shown. (**b**) Colony-forming unit assay (CFU) was performed in passage 1 and 3, and the number of colonies formed per 100 seeded cells is shown (0.75:1 n = 9, 1:1 n = 11, and 1.5:1 n = 8). No significant difference between tested variants was detected, although variant 1.5:1 tended to form less colonies in passage 3 compared to passage 1. Kruskal–Wallis One-Way Analysis of Variance on Ranks was used for statistical analysis (Trend *p* ≤ 0.1). Only significant differences within the same condition (between different time points) or within the same time point (between different conditions) are shown.

The WST1 assay can easily be used to monitor the metabolic activity of cells since only dividing cells can reduce the tetrazolium salt (Figure 4a). Within variant 0.75:1, no difference in relative absorption was detected for all tested time points. Contrastingly, for variant 1:1 and 1.5:1, an (significant) increase in metabolic activity during passage 3 was observed. No significant difference between the tested variants was seen.

The ability of the isolated UC cells to form colonies after seeding them in a low density was tested in a next step with a classical CFU assay (Figure 4b). The cells isolated with all three grinding variants formed colonies in the CFU assay to a similar extent, whereas the variability between biological replicates was higher in passage 1. The colony-forming potential tended to be lower only for variant 1.5:1 in passage 3 compared to passage 1.

### 3.4. Surface Marker Expression and Tri-Lineage Differentiation of UC Cells In Vitro

**Figure 5:**
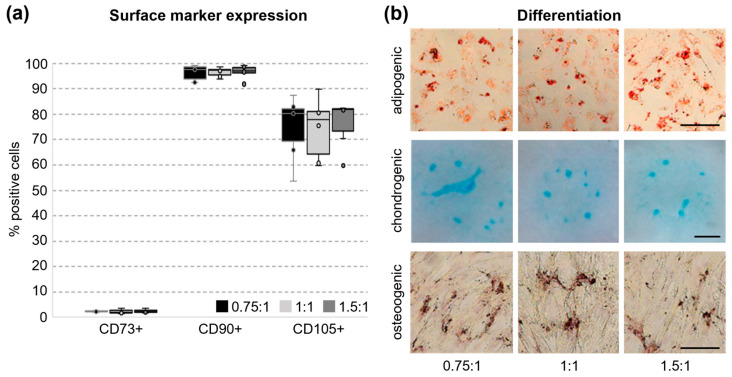
MSC-typical surface markers and lineage commitment of isolated UC cells. (**a**) The number of cells expressing the classical MSC markers CD73, CD90, and CD105 was examined via flow cytometry after 19 days of culture. The majority of cells expressed CD90 and CD105. The number of cells expressing CD73 was very low. No significant differences between tested variants were detected. One-Way Analysis of Variance (CD73+) or Kruskal–Wallis One-Way Analysis of Variance on Ranks (CD90+ and CD105+) was used for statistical analysis (n = 6). (**b**) After adipogenic, chondrogenic, or osteogenic differentiation in passage 3 or 4, UC cells were stained with Oil Red O, Alcian Blue, or Alizarin Red, respectively. The cells were able to undergo all three differentiation lines in vitro. Scale bar 200 µm.

The expression of the traditional MSC markers CD73, CD105, and CD90 was tested via flow cytometry when cells were harvested after finishing passage 0 after 19 days in culture [5]. All markers could be detected and the number of positive cells did not significantly differ between the tested grinding variants (Figure 5a). With 2.4% ± 0.6, CD73 showed the lowest number of positive cells. CD90 (96.5% ± 2.4) and CD105 (75.3% ± 10.8) were expressed on the surface of the majority of the isolated cells. As multipotency is a key feature of MSCs, we also tested for the capability to differentiate into the adipogenic, chondrogenic, and osteogenic lineage. Lipid droplets were stained with Oil Red O, proteoglycans in the chondrogenic extracellular matrix with Alcian Blue, and osteogenic calcium deposits with Alizarin Red (Figure 5b). All tested variants could be successfully differentiated into the three different lineages and, therefore, show multipotency.

## 4. Discussion

Choosing the best method with which to isolate primary cells from tissue is of the utmost importance in order to optimally use the cell source while obtaining the desired cell type in very good quality. On the one hand, enzymatic digestion methods are expensive and might favor specific cells depending on the selected enzyme or enzyme cocktail and the subsequent purification method. On the other hand, with explant cultures, the outgrowth of cells needs a longer time, especially for slow-growing cells, and require a substantial amount of individual handling of the samples, which hampers standardization. Moreover, the frequent floating of the tissue fragments in the medium can diminish the cell yield, since the cells cannot migrate to the plastic surface while floating [7]. To avoid these disadvantages, we tested a semi-automated benchtop device to isolate cells from porcine umbilical cord samples by using a mechanical approach. The automated grinding process was very fast, produced no or only minimal heat, and enhanced the reproducibility of the isolation procedure. The material still has to be cut into smaller pieces manually before loading the grinding tube, which might be a drawback for larger sample sizes. The TIGR instrument combines shearing and cutting forces for the dissociation of cells and the velocity and duration of each step can be selected [44]. For our samples, there was no need to optimize the grinding program and we could use the pre-installed program “medium”. Another advantage compared to our standard explant cultures running over 19 days is the possibility of obtaining cellular parameters (e.g., viability and number) right after tissue grinding without having to wait days or weeks until the first cell harvest.

The viability of cells was acceptable directly after isolation, even though considerably lower compared to later passages. We did not test if the shear forces during grinding lead to apoptosis, but, in previous studies, this was not the case for the TIGR device [44]. Therefore, we hypothesized that the proportion of apoptotic cells could be neglected. It seems more likely that the presence of tissue or cell debris and some remaining erythrocytes negatively influenced the number of cells stained with trypan blue. Since the viability after cell harvest in all tested passages was very high (91% versus 61% directly after isolation), the isolated cells seemed to have a very good quality. The lack of a larger number of floating cells also argues for a vital cell population.

Cell isolation with tissue grinding is performed without any preference for a specific cell type or cell characteristic [44]. Thus, the high morphological heterogeneity in passage 0 was probably due to the isolation method and reflects the well-known heterogeneity between UCC subpopulations from distinct anatomical regions of the UC having different histological and developmental characteristics [28]. Cultures from porcine UC explants were shown to first form rhomboid-shaped cells, transforming into a spindle-shaped monolayer culture [36], which was also observed in our cultures. Cells isolated from porcine WJ presented a heterogeneous morphology, as well, reaching from a spindle-like shape to a small and round shape when forming dense colonies [22]. The latter one we also observed frequently in this study (as seen in Figure 2c, passage 0, 1.5:1, left). During cultivation, the classical fibroblast-like spindle shape was documented for UC cells (from whole UC or WJ) in porcine, bovine, and human cells, which is in accordance with our study [7,22,34]

Colony formation is a characteristic of MSCs [51]. It reflects their self-renewal ability, and is a prerequisite for the long-term growth of those cells [52]. The results from CFU assays showed that cells isolated by using the TIGR system give rise to colonies, thereby confirming this MSC criterium. The donor and cell type can affect the colony-forming potential of MSCs [52]. We found a high interindividual variation, specifically in P1 of our cultures, which is typical for UC-MSC and ascribed to the higher primitivity of stem cells from perinatal tissues [19]. In addition, UC-MSC from individual donors are highly heterogeneous regarding ECM components, specifically glycoproteins, which affects functions like aging, the antioxidative capacity, and the lineage differentiation [19]. Moreover, the self-renewal potential seemed to decline until passage 3. The latter effect was described previously in MSCs derived from different tissue sources, especially within the first passages in the culture [51,53]. Interestingly, in human-UC-blood-derived cells, the CFU potential remained constant between passage 2 and 10 [54]. In human UC perivascular cells, by contrast, the ability considerably declined from passage 1 to 3, followed by stabilization over six passages and a repeated increase in colonies formed from passage 10 to 12 [55]. The long-term cultivation of the cells was beyond the scope of the present study but will be an important part of further research.

However, isolation and culture conditions might also play a role. For example, basic fibroblast growth factor (bFGF) is important for self-renewal and cell survival, e.g., by restoring telomerase activity [56]. In explant cultures, the tissue is a source of bFGF, which is missing with our method. Moreover, we added no bFGF to our growth medium thereby, possibly promoting the first steps of differentiation. To stabilize the primitive UC-MSC in vitro, a combination of media supplements published by Lim et al. [20] can be used.

The surface antigens CD73, CD90, and CD105 are claimed as typical MSC markers [57], and, thus, we tested their abundance, as well. Indeed, the surface markers CD90 and CD105 could be detected successfully, while the number of cells expressing CD73 was very low.

For the interpretation of our results, various factors, for example, species differences, the composition of the isolated cell population, and changes induced due to the in vitro culture conditions (notably the medium composition) and time (passage used), have to be mentioned [7,58,59]. Specifically, there are marked differences between the UC from humans and pigs. Most importantly, the porcine UC has remnants from the allantois and occluded yolk sack and is extensively vascularized. Its connective tissue maintains over the complete pregnancy an embryonic character, with its vasculogenic and hematopoietic activity reflected by specific mesenchymal cells with endothelial features as well as angioblasts, hemoblasts, and erythroblast [29]. Thus, only a smaller part of the connective tissue of the UC from pigs corresponds to the typical mucous connective tissue characteristic for humans [29]. In accordance with the dominance of embryonal, vascular, or young mesenchymal connective tissue, the very low presence of CD73 is not surprising. Embryonic stem cells express no CD73 and CD105, but CD90, which we found in 96.5% of our cells. A higher proportion of them is also positive for CD105 (75.3%), which fits with its role in proliferation, vascular remodeling, and angiogenesis [60,61]. CD90 is seen as a stemness-associated marker indicating a capacity for self-renewal and the maintenance of the undifferentiated cells [32,36]. This is in agreement with data showing that the CD90 expression in cultured porcine UC cells decreased from 66% in passage 4 to 24% in passage 11 (maximum: 95% in P6) [36]. Taking into account the tissue properties of the porcine UC, it cannot be expected that pUCCs are negative for hematopoietic surface markers. Instead, the angiogenic and hematogenic MSC population of the pUC could be an interesting model for investigating the initial steps in early vasculogenesis.

The spatial and temporal stability of the marker expression in UC-derived cells in different species is still under consideration and differs depending on the experimental setup [7,62]. For example, in bovine WJ-derived cells and human UC explant cells, the CD73 expression was higher in passages 5 and 6, respectively [63,64]. On the other hand, CD90 and CD105 expression was shown to decrease in human UC cells from passage 4 to 8 [62], pointing to a higher differentiation potential [32]. In addition, the marker expression is dependent on the UC compartment used for cell isolation. For example, CD73 is primarily expressed in human UC vessels, WJ, and endothelium, but not in the perivascular region [7]. CD90 is found in most human UC compartments except for the endothelial lumen lining [7].

Using a few cell surface molecules is not sufficient to fully characterize the functional potency of UCCs [5]. Therefore, we also tested for the in vitro differentiation potential of pUCCs isolated via tissue grinding. We found that cells were able to perform three-lineage differentiation, adipose, chondrogenic, and osteogenic, showing their multipotency. For UC-MSC, differences in the differentiation potential depending on the lineage are well-known [7]. Depending on the conditions and the compartment used, osteogenic differentiation might be the most problematic for UC-derived cells, as an insufficient differentiation was shown previously [7].

Besides testing for the cellular properties of pUCCs isolated with the TIGR device in general, we also aimed at comparing three variants differing in the tissue-to-medium ratio. All three variants tested in this study could be successfully used to isolate viable, proliferative UCCs. In a variety of cellular parameters, all variants gave rise to comparable results (the viability, cell size, morphology, MSC marker expression, and three-lineage differentiation). Nevertheless, differences relevant for the experimental setup were observed. The dramatically lower cell yield in variant 1.5:1 is surely the most prominent one. Furthermore, cells obtained with this variant were not able to increase their cell proliferation rate during cell cultivation. Both other variants significantly increased the cellular growth in later passages. In WJ-derived pUCCs, the proliferation rate slowed down in higher passages (P4–P11) [36]. Until passage 3, we could not confirm this, but it might occur as well during prolonged proliferation. In contrast to the other variants, cells from 1.5:1 tended to lose their ability to form colonies during cultivation. Thus, using more tissue than medium during grinding (as in 1.5:1) cannot be recommended for pUCCs.

## 5. Conclusions

In summary, tissue grinding is highly suitable for isolating high-quality cells from whole UC tissue, which show MSC-like features. For a further characterization of the possible MSC phenotype of pUCCs, additional experiments (e.g., defining pUCC subpopulations and specific surface markers, growth factor assays, and hematopoietic and endothelial marker expression) will be necessary in the future. Tissue grinding provides a scalable, fast, and reproducible way to isolate UCCs with a high yield. The cells might be used in a wide range of therapeutical and biotechnological applications, such as understanding and treating severe disorders, screening for new drugs and toxins, or tissue engineering to create surrogate tissues and organs [7,65,66], but also in cellular agriculture [16]. Cells from supposedly waste tissues like UCs will be especially useful in transplantation medicine because of their proposed lower rate of graft-versus-host disease [66].

## Data Availability

The data presented in this study are available upon request from the corresponding author.

## Abbreviations

The following abbreviations are used in this manuscript:

bFGF	basic fibroblast growth factor
CFU	colony-forming unit
CHO	Chinese hamster ovary
CV	crystal violet
ECM	extracellular matrix
HEK	human embryonic kidney
MSC	mesenchymal stromal cell
UC	umbilical cord
UCC	umbilical cord cell
WJ	Wharton’s Jelly
